# Teriflunomide and monomethylfumarate target HIV-induced neuroinflammation and neurotoxicity

**DOI:** 10.1186/s12974-017-0829-2

**Published:** 2017-03-11

**Authors:** Björn Ambrosius, Simon Faissner, Kirsten Guse, Marec von Lehe, Thomas Grunwald, Ralf Gold, Bastian Grewe, Andrew Chan

**Affiliations:** 1 Department of Neurology, St. Josef-Hospital, Ruhr-University Bochum, Gudrunstr. 56, 44791 Bochum, Germany; 2 Hotchkiss Brain Institute and Department of Clinical Neurosciences, University of Calgary, Calgary, Alberta Canada; 3 Department of Neurology, University Hospital Bern and University of Bern, Bern, Switzerland; 4 Department of Neurosurgery, Knappschaftskrankenhaus Bochum, In der Schornau 22-25, 44892 Bochum, Germany; 5 Fraunhofer Institute for Cell Therapy and Immunology, Leipzig, Germany; 6 Department of Molecular and Medical Virology, Ruhr-University Bochum, Universitätsstr. 150, 44801 Bochum, Germany

**Keywords:** HIV-associated neurocognitive disorder, HAND, Glial activation, Microglia, Neuroinflammation, Leflunomide, Dimethyl fumarate

## Abstract

HIV-associated neurocognitive disorders (HAND) affect about 50% of infected patients despite combined antiretroviral therapy (cART). Ongoing compartmentalized inflammation mediated by microglia which are activated by HIV-infected monocytes has been postulated to contribute to neurotoxicity independent from viral replication. Here, we investigated effects of teriflunomide and monomethylfumarate on monocyte/microglial activation and neurotoxicity. Human monocytoid cells (U937) transduced with a minimal HIV-Vector were co-cultured with human microglial cells (HMC3). Secretion of pro-inflammatory/neurotoxic cytokines (CXCL10, CCL5, and CCL2: *p* < 0.001; IL-6: *p* < 0.01) by co-cultures was strongly increased compared to microglia in contact with HIV-particles alone. Upon treatment with teriflunomide, cytokine secretion was decreased (CXCL10, 3-fold; CCL2, 2.5-fold; IL-6, 2.2-fold; *p* < 0.001) and monomethylfumarate treatment led to 2.9-fold lower CXCL10 secretion (*p* < 0.001). Reduced toxicity of co-culture conditioned media on human fetal neurons by teriflunomide (29%, *p* < 0.01) and monomethylfumarate (27%, *p* < 0.05) indicated functional relevance. Modulation of innate immune functions by teriflunomide and monomethylfumarate may target neurotoxic inflammation in the context of HAND.

**Electronic supplementary material:**

The online version of this article (doi:10.1186/s12974-017-0829-2) contains supplementary material, which is available to authorized users.

## Introduction

Human immunodeficiency virus (HIV)-infected patients suffer serious complications, of which the pathogenesis of HIV-associated neurocognitive disorders (HAND) is one of the least understood. Since the introduction of combined antiretroviral therapy (cART) in the early 1990s, higher prevalence of the less severe phenotypes of HAND were reported [1, 2]. One key hypothesis to explain the occurrence of HAND despite inhibition of viral replication is the “bystander hypothesis”. It postulates that microglia become activated by HIV-infected monocytes and T cells, which have entered the brain early during infection [3, 4]. As a consequence, microglia release pro-inflammatory and neurotoxic factors that induce neurotoxicity [5]. Several studies demonstrated that immune activation of monocytes and microglia contribute to neurodegeneration in the context of HAND [4, 6–9]. Earlier results of our group stressed the importance of monocyte involvement for full microglial activation. HIV-transduced monocytes act as amplifier of microglial activation and neurotoxicity [4]. We also demonstrated that a panel of microglia-derived cytokines was differentially regulated in vitro (CXCL10, CCL5, and IL-6). These cytokines were associated with markers of early neurodegeneration in cerebrospinal fluid (CSF) of HIV-infected, yet neurocognitively not symptomatic patients [4]. Thus, therapeutic modulation of innate immune cell activation may hypothetically also affect neurodegeneration in the context of HAND.

Different agents have been demonstrated to affect microglial and monocyte activation in the context of autoimmune neuroinflammation. Teriflunomide (Teri) mainly inhibits de novo pyrimidine synthesis in mitochondria by acting on dihydroorotate dehydrogenase (DHODH), which leads to inhibition of T and B cell proliferation [10]. In addition, DHODH-independent effects with decreased release of pro-inflammatory cytokines from monocytes are described [11]. Fumaric acid esters lead to the intranuclear translocation of nuclear factor 2 (Nrf2). They enhance the expression of anti-oxidative enzymes and decrease pro-inflammatory cytokine secretion by microglia during experimental autoimmune neuroinflammation [12, 13]. In HIV-infected macrophages in vitro, monomethylfumarate (MMF) decreases pro-inflammatory cytokine release and induces an antioxidant response [14, 15]. However, the effect of either Teri or MMF on monocyte/microglia interaction in the context of HAND remains elusive. Here, we set out to investigate the role of Teri and MMF in the context of microglial activation and neurotoxicity triggered by HIV-infected monocytes.

## Methods

### Cell culture

Human microglial cell line 3 (HMC3, Dr. J. Pocock, University College London) was produced by transfecting human embryonic brain-derived macrophages with the large T antigen of the simian virus 40. The cell line expresses microglial and macrophage surface markers [16]. Similar to primary microglia, these cells show a distinct response of cytokines and chemokines in contact to pathogens [17] and were already described in the context of HIV [18]. Cells were cultured in Minimum Essential Media (MEM) (Thermo Fisher Scientific, Darmstadt, Germany), supplemented with 10% fetal calf serum (FCS) (Sigma-Aldrich, Taufkirchen, Germany) and 100 units/ml (U/ml) penicillin/streptomycin (Pen/Strep, Invitrogen, Darmstadt, Germany) in T-75 flasks (PRIMARIA™ Tissue Culture Flask, Becton Dickinson, Heidelberg, Germany). Cells were passaged at a confluence of 90%. For experiments, cells were plated in 96-well plates (10,000 cells/well) (Sarstedt, Nümbrecht, Germany) 24 h before co-culture experiments or treatment with pharmacological substances.

Primary human microglia were isolated from patients with intractable epilepsy, as previously described [19]. Cells were plated at the same density as HMC3 cells.

U937 cells (further referred to as monocytoid cells, Sigma Aldrich, München, Germany) were derived from a patient with generalized histiocytic lymphoma [20]. Cells were cultured in RPMI-1640 (Thermo Fisher Scientific), supplemented with 10% FCS and 100 U/ml Pen/Strep. For transduction, 50,000 monocytoid cells were seeded in 24-well plates (Sarstedt, Nümbrecht, Germany) and incubated with HIV particles as previously described [4].

Human fetal neurons (HFN) were isolated from 18–20-week-old brains that were obtained from therapeutically aborted fetuses as previously described [21]. Cells were plated in MEM supplemented with 10% fetal bovine serum, 1 μM sodium pyruvate, 10 μM glutamine, ×1 non-essential amino acids, 0.1% dextrose and 1% penicillin/streptomycin (HFN-complete medium; Invitrogen, Burlington, Canada). Cells were plated in poly-L-ornithine coated (10 μg/ml) T75 flasks (5 × 10^7^ cells in 25 ml media) and treated with three cycles of 25 μM cytosine arabinoside (Sigma-Aldrich, St. Louis, MO) to kill dividing astrocytes. For experiments, HFN were plated in coated 96-well plates (100,000 cells/well in 100 μl medium). After 48 h media was changed to MEM media supplemented with 1% Pen/Strep for 5 h. At this point, the media was removed and cells were treated with conditioned media of HMC3 cells or HMC3/monocytoid cells co-cultures. After 48 h, cells were stained with propidium iodide (PI, 1 μg/ml; Sigma-Aldrich), fixed with 4% PFA, and stored in PBS at 4 °C.

### Preparation of viral particles and transduction of target cells

All necessary transduction controls for the investigation of the role of monocytoid cells in contact with viral particles were investigated in detail by our group previously [4]. Different viral particles which were either not able to fuse with monocytoid cells (“HIV-fusion-deficient”), did not contain viral RNA (“HIV empty”), or were deficient of viral enzymes (“HIV-pol-deficient”) were employed to delineate the essential steps of microglial/monocyte activation. In particular, viral particles which consisted of gag but which did not contain viral RNA were used to exclude that the process of transduction is responsible for activation and neurotoxicity (“HIV empty”) [4].

Preparation of viral particles after transfection of HEK293T cells as well as characteristics of the HIV vector have been described previously [4]. Transfections were conducted with the calcium-phosphate co-precipitation method [22]. As an additional control for HIV vector particles, supernatants of HEK293T cells treated with transfection reagent were used. Viral particles were produced as self-inactivating HIV particles [23] and contain HIV RNA with enzymatic equipment for reverse transcription and integration into the genome of monocytoid cells [4]. For this, HIV CS-CG was co-transfected with HGP^syn^, pcRev, pcTat, and pseudotyped with pHIT-G (further referred to as HIV vector). HIV CS-CG encodes for a minimal HIV genome, which is packed into the viral particles and contains a GFP-sequence [4].

Due to the time needed for viral gene expression in host cells [24], transduction efficiency was analyzed by flow-cytometry 48 h after transduction based on the number of GFP-positive cells. Transduction of monocytoid cells with the HIV vector led to consistent transduction rates of 4–6% (4.3 ± 0.21 (mean ± SEM, *n* = 3)) similar to the rate of HIV-infected monocytes in the CNS of infected patients [25].

Viability of monocytoid cells was determined via FACS using 7AAD (eBioscience, Frankfurt a. M., Germany, 0.5 μl/50,000 cells). Viability of HMC3 was determined using 4 μg/ml bisBenzimide H 33342 (Sigma-Aldrich, Taufkirchen, Germany) for 2 h, followed by 7AAD (0.5 μl/well). Mean fluorescence intensity (IX51, Olympus, Hamburg, Germany) was analyzed using ImageJ (NIH, Bethesda, USA).

### Co-culture and pharmacological treatment

HIV vector-transduced monocytoid cells were treated with Teri (10 and 30 μM) or MMF (10, 30, and 100 μM) dissolved in dimethlysulfoxide (DMSO) immediately before the application to microglia in a 1:2 ratio. This experimental design was chosen to ensure that the integrity of the microglial cell layer was not influenced by additional pipetting steps which may alter activation status. The cells were co-cultured with either Teri or MMF for 24 h. Supernatants were collected, centrifuged (4000 rpm, 5 min) and stored at −80 °C for further analysis. Supernatants of untreated co-cultures served as controls. To investigate an effect of pharmacological agents exclusively on microglia, microglia in the presence of viral particles were treated with Teri or MMF.

### Cytokine Bead Array

Cytokine secretion by the monocytoid cell/microglial co-culture was analyzed using the FACS-based Cytokine Bead Array (CBA) (Becton Dickinson, Franklin Lakes, USA; FACS Canto II). Selection of cytokines followed our previous study where CXCL10, CCL5, CCL2, and IL-6 were differentially regulated upon co-culture of HIV vector-transduced monocytoid cells with microglia. Furthermore, CXCL10, CCL5, and IL-6 correlated with neurofilament heavy chain in the CSF of HIV^+^ patients [4]. In addition, we also analyzed IFN-γ, IL-1β, IL-4, and IL-10 (500 events per cytokine). Further analysis was performed using the software FCAP Array v.3.

### Immunocytochemistry and microscopy of HFN

After PI staining and PFA fixation, immunofluorescence staining was performed using blocking buffer for 1 h. Incubation with anti-microtubuli associated protein-2 (MAP-2) primary antibody (dilution 1:1000; Sigma, Oakville, Canada) overnight (4 °C), followed by Alexa Fluor 488 (dilution 1:250, Invitrogen, Burlington, Canada), and staining of nuclei with Hoechst S769121 was performed thereafter. Images were taken at ×10 magnification (ImageXpress®, Molecular Devices, Sunnyvale, CA). Analysis was performed using MetaXpress® with the algorithm “multiwavelength cell scoring” and data from nine sites/well were averaged to one data point. Dead neurons will not adhere after fixation, and thus, the number of remaining neurons correlates with cell death [26]. To correct for adherent, but dead neurons, the few adherent PI-positive neurons (MAP-2^+^PI^+^; 0.1% in average of all conditions) were subtracted from MAP-2^+^PI^−^ cells, representing surviving neurons. H_2_O_2_ (3 μM) was used as a positive control to induce cell death in neurons.

### Statistical analysis

Experiments were performed in triplicates, if not otherwise stated. Data were statistically analyzed using a parametric one-way ANOVA with post hoc analyses as indicated in the figure legends. Statistical significance was shown as *p < 0.05; **p < 0.01; ***p < 0.001; and ****p < 0.0001 (GraphPad Prism v.7, GraphPad Software, USA).

## Results

### Highest secretion of pro-inflammatory and neurotoxic cytokines occurs only after contact of microglia with HIV-infected monocytoid cells

We first investigated if HIV-transduced monocytoid cells are mandatory for broad microglial activation or whether microglial contact with HIV vector without involvement of monocytes would suffice. As depicted in Additional file 1: Figure S1, the strongest secretion of CXCL10, CCL5, CCL2, and IL-6 was found in microglia in direct contact with HIV-transduced monocytoid cells (*p* < 0.001). In comparison, all control conditions (HIV-transduced monocytoid cells alone, microglia alone with HIV vector, co-culture of microglia with non-infected monocytoid cells, Additional file 1: Figure S1) showed significantly lower cytokine release. HMC3 microglia in contact with HIV vector alone showed higher release of CXCL10 and IL-6 than HMC3 microglia alone (CXCL10 *p* < 0.001; IL-6 *p* < 0.001). CCL5 and CCL2 were not altered. Thus, in line with our previous data [4], the strongest chemokine/cytokine secretion occurred only after contact of microglia with HIV-infected monocytoid cells but not with viral particles alone. Secretion of CXCL10, CCL5, CCL2, and IL-6 was differentially regulated. Other cytokines (IFN-γ, IL-1β, IL-4, and IL-10) were not detected (data not shown).

### Teri and MMF reduce activation of monocyte/microglia co-culture

**Fig. 1:**
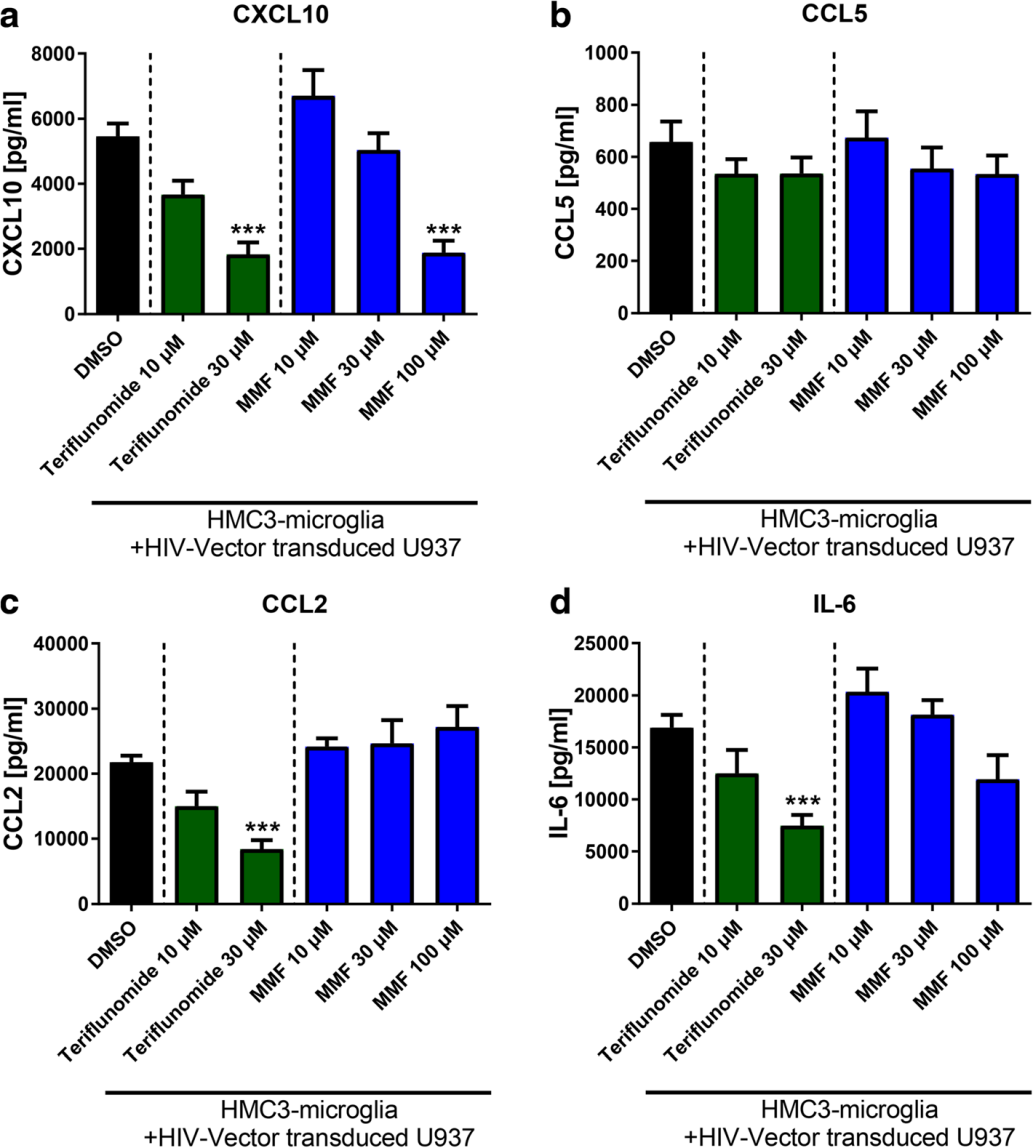
Teri and MMF reduce cytokine secretion of monocyte/microglia co-culture. Treatment of the co-culture of HMC3 microglial cells with HIV vector-transduced monocytoid cells (U937) with Teri or MMF. Shown are three independent experiments performed in triplicates. Significance is observed in comparison to microglia in co-culture with HIV vector transduced monocytoid cells. DMSO was used as a solvent and used in the control condition HIV vector. Data are depicted as mean ± SEM. **a**, **c,** and **d**: Statistical analysis was performed using one-way ANOVA (<0.0001) with Tukey’s multiple comparisons test as post hoc analysis. **b** No significant difference. ***p < 0.001

Next, we examined whether Teri and MMF can modulate the cytokine secretion in the co-culture setting. Teri decreased the cytokine secretion in microglia exposed to HIV vector-transduced monocytoid cells in a dose dependent fashion (Teri 30 μM, CXCL10; 3-fold, CCL2; 2.5-fold, IL-6; 2.2-fold; *p* < 0.001) (Fig. 1a–d), whereas CCL5 was not altered (B). One hundred microliter MMF decreased secretion of CXCL10 (2.9-fold; *p* < 0.001) (Fig. 1a) but did not alter the release of CCL5, CCL2, and IL-6 (Fig. 1b–d). DMSO used as solvent did not have an effect on cytokine release (data not shown). Teri (30 μM) or MMF (100 μM) in concentrations used for the co-culture experiments did not induce cell death in monocytoid cells or microglia (Additional file 2: Figure S2).

Similar, albeit restricted effects of pharmacological treatment, were also observed in the absence of monocytoid cells. In microglia in contact with HIV vector particles, treatment with 100 μM MMF led to a reduction of CXCL10 (4.6-fold) and IL-6 (2.8-fold, all *p* < 0.001; Additional file 1: Figure S1A and D). In contrast, 30 μM Teri did not alter the secretion of CXCL10, CCL5, and IL-6 (Additional file 1: Figure S1).

**Fig. 2:**
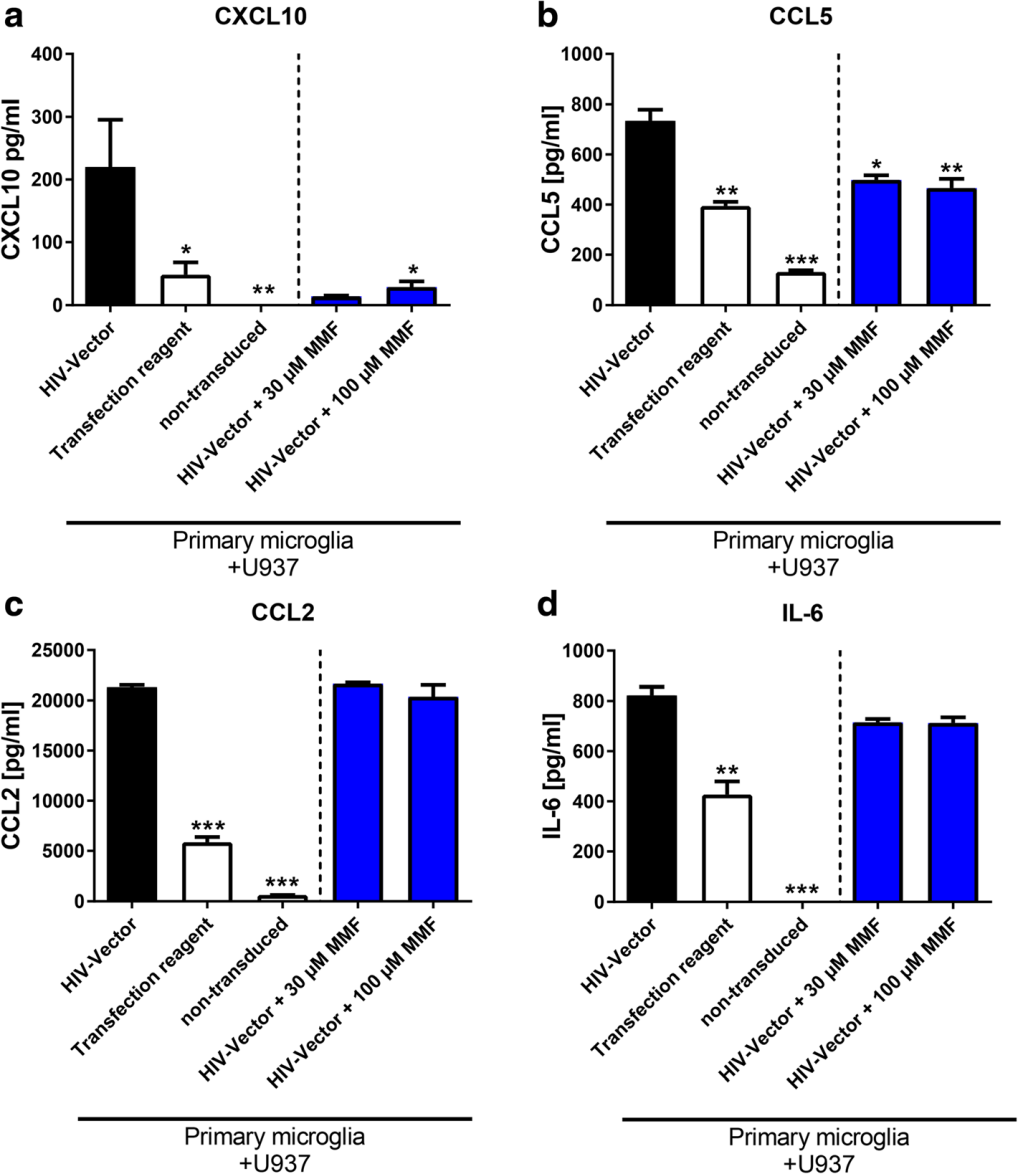
Reduced cytokine secretion in primary microglia co-culture upon treatment with MMF. MMF treatment of primary microglia in co-culture with HIV vector-transduced monocytoid cells reduced the release of CXCL10 and CCL5 (**a**, **b**), whereas secretion of CCL2 and IL-6 were not altered (**c**, **d**) . Monocytoid cells (U937) were added after transduction (HIV-Vector) or non-transduced (*non-transduced*) to primary microglia. Transfection reagent was used as additional control. Shown are one to two experiments in duplicates or triplicates. Significant differences are observed in comparison to microglia in co-culture with HIV vector-transduced monocytoid cells (***). Data are presented as mean ± SEM. Statistical analysis was performed using one-way ANOVA (<0.0001) with Tukey’s multiple comparison test as post hoc analysis. *p < 0.05; **p < 0.01; ***p < 0.001

To rule out that the effects were restricted to HMC3 microglia, data were further corroborated using primary human microglia with HIV vector-transduced monocytoid cells (Fig. 2). Similar to experiments performed with HMC3 microglia, the co-culture of primary microglia with HIV vector transduced monocytoid cells showed higher secretion of CXCL10 (*p* < 0.01), CCL5, CCL2, and IL-6 (all *p* < 0.001) compared to non-transduced control conditions. Also, the control condition with the transfection reagent alone confirmed that the transfection reagent was not responsible for activation (CXCL10 *p* < 0.05; CCL5 *p* < 0.01; CCL2 *p* < 0.001; and IL-6 *p* < 0.01 in comparison to HIV vector). Treatment with MMF (100 μM) reduced CXCL10 (7.7-fold, *p* < 0.05; Fig. 2a) and CCL5 secretion (1.6-fold, *p* < 0.01; Fig. 2b) whereas CCL2 and IL-6 were not altered (Fig. 2c, d). Therefore, data generated using HMC3 mimic the response of primary microglia.

### Supernatants of Teriflunomide and MMF treated monocyte/microglia co-cultures reduce HIV-mediated neurotoxicity

**Fig. 3:**
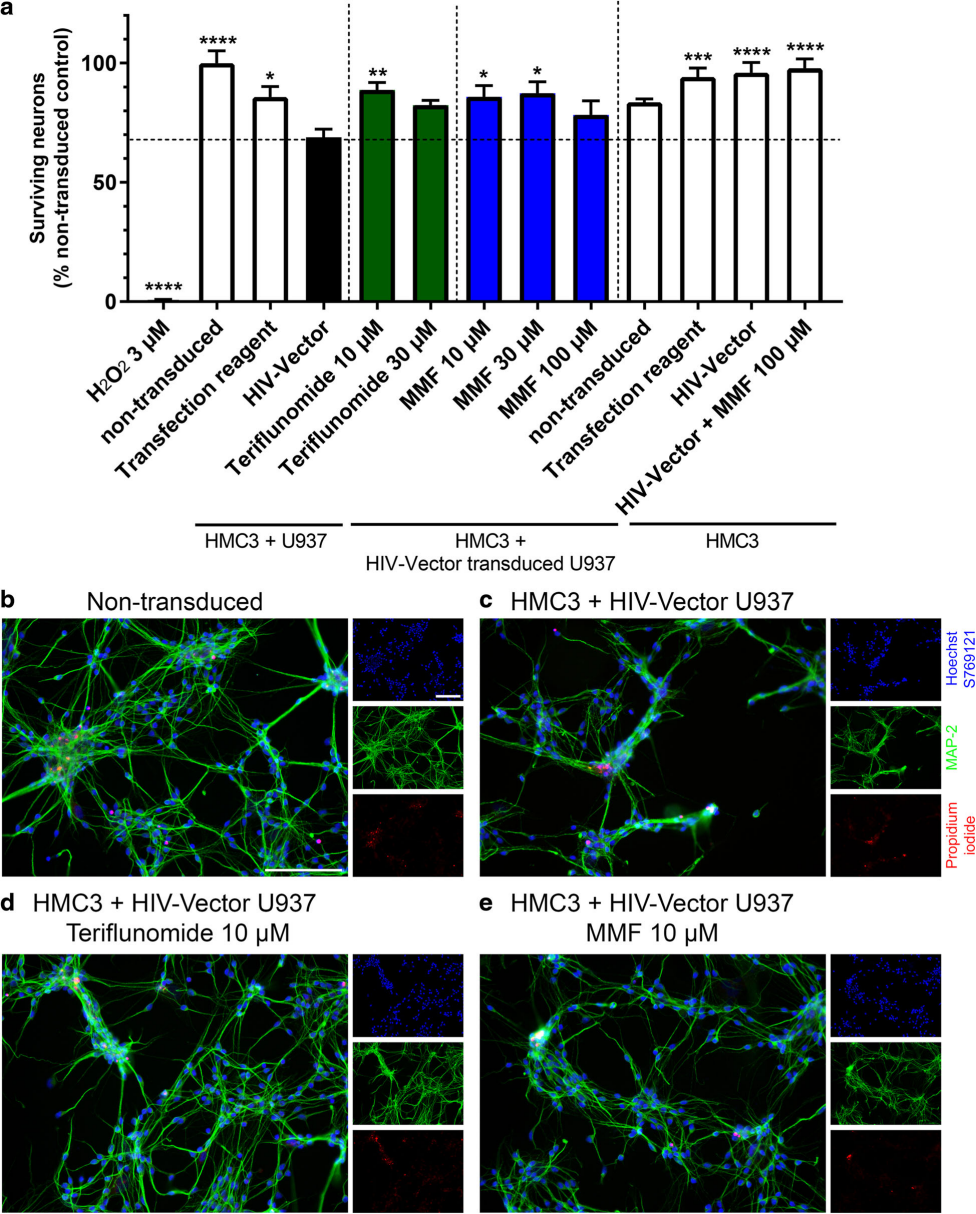
Teri and MMF preserve neuronal viability. Treatment of human fetal neurons (HFN) with conditioned media of HMC3 microglia co-cultured with HIV vector-transduced monocytoid cells induced cell death after 48 h (**a**, **c**) compared to the non-transduced control condition (*p* < 0.0001; (**b**)). Neurotoxicity was significantly reduced upon treatment with 10 μM Teri (*p* < 0.01) (**d**) and 10 μM (*p* < 0.05) (**e**) or 30 μM MMF (*p* < 0.05). Staining for microtubuli-associated protein (MAP)-2 (*green*), nuclei (Hoechst S769121, *blue*) and propidium iodide (*red*). Pictures are shown in ×20 magnification, the *scale bar* represents 100 μM. Shown are data generated with conditioned media of three independent experiments performed in triplicates (**a**). Statistics was performed using one-way ANOVA with Sidak´s multiple comparisons test as post hoc analysis. MAP-2^+^PI^+^ cells were subtracted from MAP-2^+^PI^−^ cells, thus only showing surviving neurons. Data are normalized to the control condition HMC3 + non-transduced monocytoid cells and are shown as mean ± SEM. Significance is shown compared to conditioned media of the HIV vector transduced co-culture condition (“HIV vector”). *p < 0.05; **p < 0.01; ***p < 0.001; ****p < 0.0001

To investigate if pharmacological treatment also has functional effects on neuronal viability, we analyzed monocyte/microglia-induced neurotoxicity using human fetal neurons (HFN). Neither Teri nor MMF altered neuronal viability (data not shown). Supernatants from microglia exposed to HIV vector in the absence of monocytoid cells did not elicit neuronal cell death (Fig. 3a). In contrast, supernatants derived from co-culture of microglia with HIV vector-transduced monocytoid cells strongly induced cell death after 48 h (31% fewer surviving neurons than microglia with non-transduced monocytoid cells, *p* < 0.0001; Fig. 3a, c). Treatment with 10 μM Teri (11.2% fewer neurons, *p* < 0.01), 10 μM MMF (14.1% fewer neurons, *p* < 0.05) and 30 μM MMF (12.6% fewer neurons, *p* < 0.05) led to significantly enhanced neuronal viability in comparison to supernatants derived from the co-culture of microglial cells with HIV vector transduced monocytoid cells. Higher concentrations of Teri (30 μM) and MMF (100 μM) did not result in increased neuronal viability. H_2_O_2_ (3 μM) was used as positive control and induced complete neuronal cell death.

## Discussion

In this study, we investigated the effect of Teri and MMF in the context of HIV-mediated monocyte/microglial inflammation due to their well characterized anti-inflammatory properties. The ultimate goal was to reduce inflammation-related neurotoxicity. We demonstrate that Teri and MMF lead to reduced chemotactic and pro-inflammatory cytokine secretion in a co-culture system of microglia with HIV-transduced monocytoid cells. This was associated with reduced neurotoxicity of supernatant in human fetal neurons.

One limitation of this study is the use of monocytoid and microglial cell lines. However, our experiments performed with primary adult human microglia corroborated data generated using the HMC3 microglia cell line. Also, similar to the results obtained with primary embryonic microglia [4], HMC3 secrete more CXCL10, CCL5, CCL2, and IL-6 in contact with HIV vector-transduced monocytoid cells than after contact with HIV particles alone. This finding as well as lack of neurotoxicity of microglia exposed to HIV particles without monocytes is in line with the bystander hypothesis.

Microglial activation in our experimental setting is highly regulated. Mechanistically, viral RNA in monocytoid cells is required for full activation and subsequent neuronal cell death [4]. Inhibition of subsequent steps following insertion of viral RNA into monocytoid cells did not reduce microglial activation [4]. We also excluded that the process of transduction itself is responsible for activation. This agrees with findings that cART treatment does not downregulate cytokine secretion in the CNS of HIV-infected patients [27].

Enhanced CNS inflammation in HIV infection with elevated concentrations of mainly pro-inflammatory cytokines and chemokines is described in patients with HAND [7]. Differentially regulated cytokines investigated in this study are associated with the neurodegenerative markers neurofilament heavy and light chain in CSF of HIV-infected patients with and without neurocognitive impairment. This suggests ongoing inflammation with clinically silent neurodegeneration already during early stages of the disease [4, 9].

Our experimental setting does not allow us to distinguish whether treatment effects are related to interactions with microglia, HIV-transduced monocytes or both. Both Teri and MMF have anti-inflammatory effects, but with different targets. However, it remains difficult to draw definite conclusions as both agents may have a differential impact in vivo. Rather, our work suggests that therapeutic modulation of innate immune cell function using Teri or MMF may have an impact on inflammation and neurodegeneration in the context of HIV infection independent from viral replication. Teri shows a more complete reduction of cytokines CXCL10, CCL2, and IL-6 in the co-culture situation whereas MMF predominantly reduces microglia CXCL10 and IL-6 release in the absence of monocytoid cells. CXCL10 and IL-6 are associated with immune activation and crucial for general recruitment of immune cells [28, 29]. CCL2 showed a distinct effect of recruiting HIV-infected leukocytes across the BBB [30]. However, it remains speculative why higher concentrations of both Teri and MMF strongly reduced cytokine secretion but failed to further enhance neuronal viability. Neurodegeneration during HAND is presumably mediated by pleiotropic mechanisms. Our main hypothesis is that activation of innate immune cells is linked with neurodegeneration, independent from viral replication. Therefore, we used secretion of different inflammatory/neurotoxic cytokines described in the pathogenesis of HAND as markers of activation of innate immune cells. Neurotoxicity assays were employed to demonstrate the functional impact of the cellular activation. In our previous work, we were able to show that cytokines differentially regulated in our model are correlated with neurofilament heavy chain as a marker for neurodegeneration in HIV^+^ patients [4]. However, this does not suggest that cytokines investigated are exclusive mediators of neuronal cell death. Thus, it remains speculative why the higher concentrations of Teri and MMF which were able to reduce cytokine secretion failed to enhance neuronal viability. Rather, our previously published results in conjunction with our present data argue for multifactorial mechanisms, which have anti-inflammatory effects and act beneficially on neuronal survival. Both Teri or MMF act on microglia and monocytes via the inhibition of nuclear factor kappa-light-chain-enhancer (NF-κB) [31, 32]. It was postulated that Teri reduces mRNA production of pro-inflammatory factors matrix metalloproteinase (MMP) 2 and MMP9 in monocytes [33]. In addition to the inhibition of mitochondrial DHODH, Teri decreased the release of IL-6 and CCL2 from activated monocytes in vitro, presumably in a DHODH independent manner [11]. Also antiviral properties of Teri have been described, hypothetically mediated by non-specific pyrimidine depletion [34, 35]. Teri has also an inhibitory effect on the expression of pro-inflammatory IL-6 in the context of Enterovirus 71 infection of the CNS cell line SY-SH5Y [33].

Data from experimental autoimmune encephalomyelitis indicate effects of MMF on microglia to be mainly mediated through activation of hydroxycarboxylic acid receptor 2 (HCAR2), leading to a phenotypic change of microglia with neuroprotective properties [13]. This was also supported by findings in a neuropathy model in rats, in which MMF causes a phenotypic shift from pro-inflammatory to anti-inflammatory macrophages [36]. However, we did not observe a phenotype change in our model based on secretion of IL-4 and IL-10. In HIV-infected macrophages, MMF upregulates heme oxygenase-1 and reduces glutamate release with reduced neurotoxicity [14]. Furthermore, it has been reported that MMF reduces cART-mediated neurotoxicity in pigtail macaques and rats [37].

Whereas we aimed at investigating effects of well-characterized agents approved for neuroimmunological disease on innate immune cells in the context of HAND, both agents used target lymphocytes. In addition, clinical relevance of these medications might be restricted due to adverse drug reactions in combination with cART (e.g., pancytopenia and hepatotoxicity). However, also HIV-infected T cells are implied in the pathogenesis of HAND (e.g., IFN-γ expressing CD8^+^ T cells) [38], clearly arguing for complex neuroimmunological interactions. In a first short clinical trial teriflunomide did not lead to a detectable decrease of CD4^+^ or CD8^+^ cells in cART untreated HIV^+^ patients [39]. Immunotherapy might especially be feasible in patients with high CD4^+^ cell counts, which can be achieved with early initiation of sufficient antiretroviral therapy [40].

Targeting enhanced inflammation in the context of HAND is promising, considering that this chronic immune activation is not eliminated by cART, which instead acts by lowering viral load [8]. Effects of Teri and MMF on cytokine levels may have implications for subsequent recruitment of inflammatory cells to the CNS [28, 41–43] and aggravation of neurodegeneration [44, 45]. Besides its function in recruiting cells and establishing an inflammatory environment, high levels of IL-6 cause sleep onset insomnia [46, 47], which could in part explain asymptomatic neurocognitive impairment in HAND [48].

In addition to targeting cytokine secretion, other therapeutic mechanisms have been proposed in the context of HAND. Treatment with FK506 has been shown to reduce mitochondrial injury and neurodegeneration in gp120 transgenic mice [49]. Another approach is the modulation of monocytoid cells via statin-treatment. Statins reduce expression of CD163, which has been related to neurotoxicity in HAND and also reduces secretion of the chemoattractant CCL2 [50].

New treatment approaches are urgently needed to attenuate HAND with its potentially devastating impact on quality of life [51]. It is expected that HAND will in future pose a high socioeconomic burden due to higher life expectancy of HIV-infected patients and increase of severity of HAND over time. Our study demonstrates that inflammatory mechanisms of innate immunity known to be involved in neurodegeneration can be modulated by agents approved in autoimmune neuroinflammation, leading to reduced neurotoxicity. Further research is warranted to understand molecular mechanisms involved in HAND with the goal to better target compartmentalized inflammation and neurodegeneration.

## Additional files

Additional file 1: Figure S1. Co-culture secretion of cytokines is elevated compared to mono-culture secretion. The co-culture of microglial cells with HIV vector-transduced monocytoid cells (left side of the panels) induced a more pronounced release of CXCL10, CCL5, CCL2, and IL-6 compared to the microglial/monocytoid mono-culture. Distinct from treatment in co-culture, MMF significantly decreased release of CXCL10 and IL-6 in HMC3 mono-culture treated with HIV vector whereas treatment with 30 μM Teri had no effect. Shown are three to six independent experiments performed in triplicates. Significance is shown in comparison to HMC3 in co-culture with HIV vector-transduced monocytoid cells (***) or in comparison to HMC3 mono-culture with HIV vector (+++) or in comparison to U937 mono-culture with HIV vector (##). Data are shown as mean ± SEM. Statistical analysis was performed using one-way ANOVA (<0.0001) with Tukey’s multiple comparison test as post hoc analysis. **p* < 0.05; **/##*p* < 0.01; ***/+++*p* < 0.001. (TIF 799 kb)
Additional file 2: Figure S2. Cytotoxic potential of Teri and MMF on monocytoid cells and microglial cells. Treatment of monocytoid cells (A) or microglial cells (B) did not lead to cell death using concentrations of up to 30 μM of Teri and up to 100 μM MMF in monocytoid cells (A) or up to 100 μM Teri and 1000 μM MMF in microglial cells (B). Treatment was performed for 24 h before analysis. Cell death was determined using 7-Aminoactinomycin D (7AAD) in FACS analysis (A) or Hoechst/7AAD co-staining (B). Three independent experiments performed in triplicates. Significance is shown in comparison to untreated monocytoid cells (A) or untreated HMC3 cells (B). Data are shown as mean ± SEM. Statistical analysis was performed using one-way ANOVA (<0.0001) with Tukey’s multiple comparison test as post hoc analysis. ****p* < 0.001 (A). For HMC3, ANOVA showed no difference (B). (TIF 259 kb)

## Abbreviations

cART: Combined antiretroviral therapy
CSF: Cerebrospinal fluid
DHODH: Dihydroorotate dehydrogenase
DMSO: Dimethylsulfoxide
FCS: Fetal calve serum
HAND: HIV-associated neurocognitive disorder
HCAR2: Hydroxycarboxylic acid receptor 2
HFN: Human fetal neurons
HIV: Human immunodeficiency virus
HMC3: Human microglial cell line 3
MAP-2: Microtubule-associated protein-2
MEM: Minimal essential media
MMF: Monomethylfumarate
MMP: Matrix metalloproteinase
NFH: Neurofilament heavy chain
NFκβ: Nuclear factor kappa-light-chain-enhancer
Nrf2: Nuclear factor 2
Pen/Strep: Penicillin/streptomycin
Teri: Teriflunomide
