# Supplementary figures and images for: Teriflunomide and monomethylfumarate target HIV-induced neuroinflammation and neurotoxicity
Source: J Neuroinflammation. 2017 Mar 11;14:51. doi: 10.1186/s12974-017-0829-2 (PMC5346211; doi:10.1186/s12974-017-0829-2)

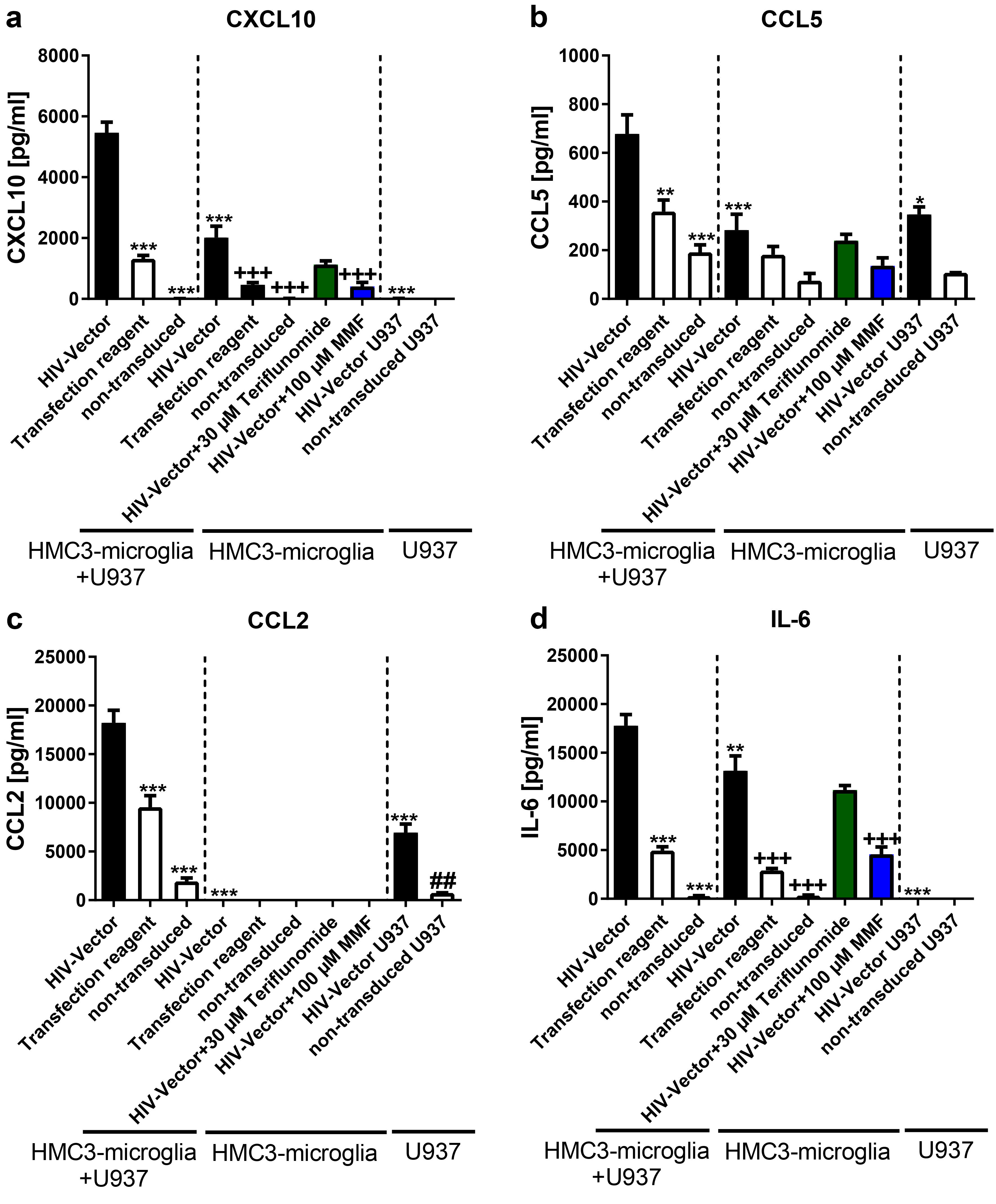

Supplement: Additional file 1: Figure S1. — Co-culture secretion of cytokines is elevated compared to mono-culture secretion. The co-culture of microglial cells with HIV vector-transduced monocytoid cells (left side of the panels) induced a more pronounced release of CXCL10, CCL5, CCL2, and IL-6 compared to the microglial/monocytoid mono-culture. Distinct from treatment in co-culture, MMF significantly decreased release of CXCL10 and IL-6 in HMC3 mono-culture treated with HIV vector whereas treatment with 30 μM Teri had no effect. Shown are three to six independent experiments performed in triplicates. Significance is shown in comparison to HMC3 in co-culture with HIV vector-transduced monocytoid cells (***) or in comparison to HMC3 mono-culture with HIV vector (+++) or in comparison to U937 mono-culture with HIV vector (##). Data are shown as mean ± SEM. Statistical analysis was performed using one-way ANOVA (<0.0001) with Tukey’s multiple comparison test as post hoc analysis. *p < 0.05; **/##p < 0.01; ***/+++p < 0.001. (TIF 799 kb) [file 12974_2017_829_MOESM1_ESM.tif]

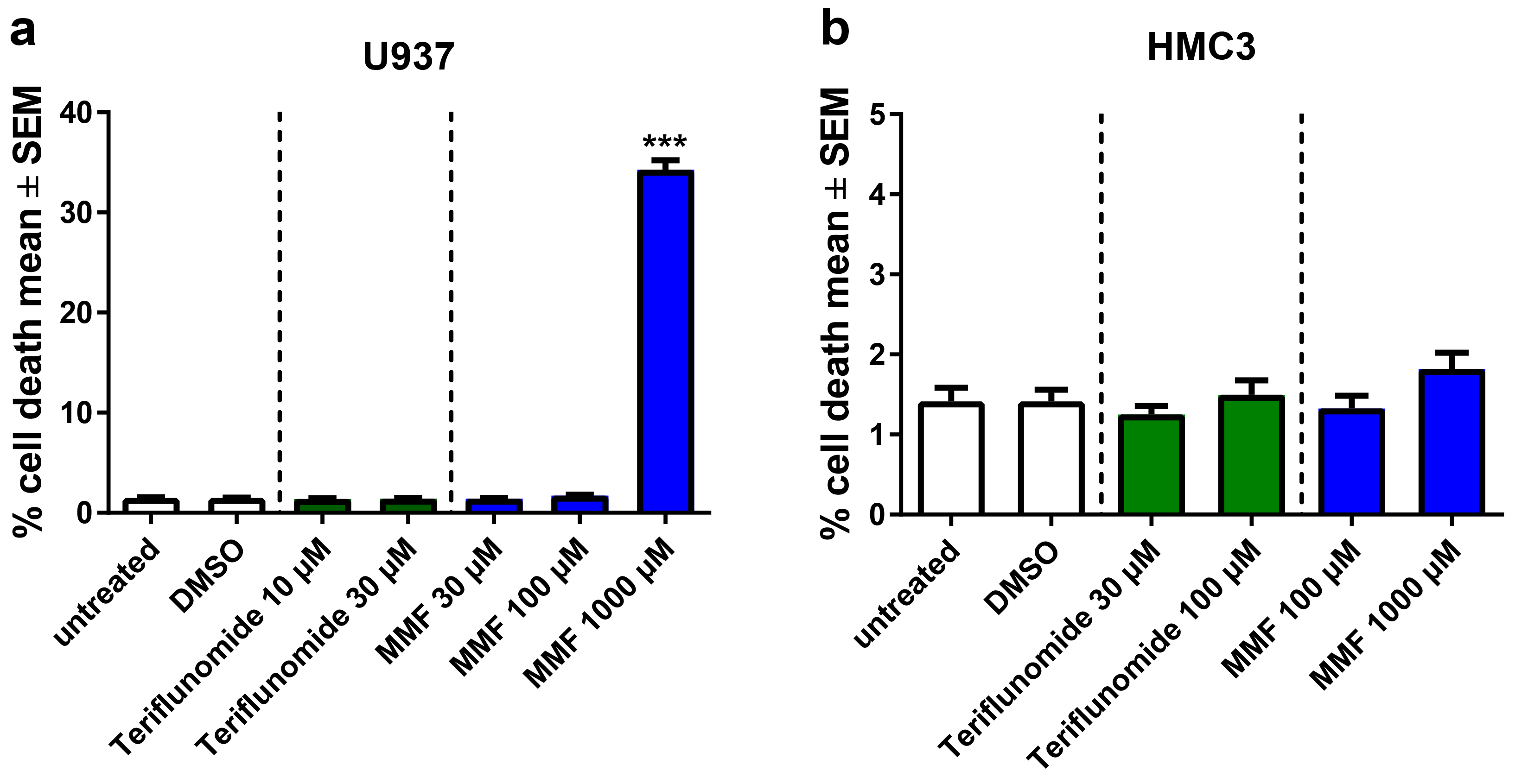

Supplement: Additional file 2: Figure S2. — Cytotoxic potential of Teri and MMF on monocytoid cells and microglial cells. Treatment of monocytoid cells (A) or microglial cells (B) did not lead to cell death using concentrations of up to 30 μM of Teri and up to 100 μM MMF in monocytoid cells (A) or up to 100 μM Teri and 1000 μM MMF in microglial cells (B). Treatment was performed for 24 h before analysis. Cell death was determined using 7-Aminoactinomycin D (7AAD) in FACS analysis (A) or Hoechst/7AAD co-staining (B). Three independent experiments performed in triplicates. Significance is shown in comparison to untreated monocytoid cells (A) or untreated HMC3 cells (B). Data are shown as mean ± SEM. Statistical analysis was performed using one-way ANOVA (<0.0001) with Tukey’s multiple comparison test as post hoc analysis. ***p < 0.001 (A). For HMC3, ANOVA showed no difference (B). (TIF 259 kb) [file 12974_2017_829_MOESM2_ESM.tif]
